# Supplementary material for: Osteoporosis induced by cellular senescence: A mathematical model
Source: PLoS One. 2024 May 28;19(5):e0303978. doi: 10.1371/journal.pone.0303978 (PMC11132490; doi:10.1371/journal.pone.0303978)
Supplement: Attachment — Submitted filename: Response to Reviewers_Osteoporosis.pdf [file pone.0303978.s002.pdf]

Response to Reviewer 1

Comment 1

This is an interesting mathematical modeling study on age-related bone loss and the role of senescent cells. There are clearly some broad assumptions made in Figure 1, but probably these are reasonable and the resulting models do seem to fit empirical data.

I don't really have expertise to evaluate the mathematics underlying the models.

However, from a biological perspective - in the abstract and throughout the paper - the authors refer to the "proliferation rate" of senescent cells. Note that part of the definition of a senescent cell is growth arrest. These cells are likely being formed and potentially being eliminated at variable rates by the immune system, with a net accrual as people age. So instead of proliferation rate, do the authors mean net formation rate (or something along those lines?) Using "proliferation rate" is not correct in this context.

Response. We thank the Reviewer the comment and we agree that using "proliferation rate" in the context of senescent cells formation may be confusing. We have now rephrased by using "net formation rate" when referring to the formation of senescent cells throughout the paper. We have also added the description of the parameter η in the table of parameters (Table 2). Changes are in blue.

Comment 2

A second point on page 3 is that it is unclear what the authors mean by deficiency of androgen receptors leading to estrogen deficiency. Note also that most evidence now indicates that the majority of the skeletal effects of androgens even in men are mediated via conversion to estrogen and via the estrogen receptor (for a review, see PMID: 28710257). They should perhaps factor this into their models.

Response. We thank for the Reviewer for the comment. We agree with the Reviewer and have now added a note in Page 3, Paragraph 6 (in blue): "Note, however, that estrogen deficiency may lead to reduced bone mass through a different mechanism than loss of the androgen receptor [44]."

Response to Reviewer 2

This is a very interesting paper. Authors attempted to consider positive and negative factors of bone formation and osteoporosis as well as cell senescence using mathematical modeling methods. The results were able to predict bone loss according to aging as well as benefit of the senolytic drug. However, I think the paper sections need to be reorganized according to Journal format.

Comment 1

a. Some of the introduction parts can be moved to “methods and modeling“ section.
b. Figure 1 should be in modeling section and serve as hypothesis of the mathematics modeling.

Response. We thank the Reviewer for the comments. We have now reformatted the article as per the Journal style. We have also included Methods and Modeling section as suggested, and included Figure 1 and Table 6 (now Table 2) in the Modeling section.

c. The conclusion reads like discussion. Authors should consider adding a discussion section and only put the summary of this paper as a conclusion.

Response. We have now renamed our former Conclusion “Discussion”, and added a “Conclusion” that itemizes the major findings of our work. The Conclusion now reads as follows:

Conclusion
In this paper we developed a mathematical model of aging bone with emphasis on the effect of senescence on loss of bone density:

Our model simulations of bone density loss for aging people are in agreement with standard charts for normal healthy men and women.

We used the model to estimate the effect of a senolytic drug, a drug which eliminates senescent cells, on reducing bone density. We quantified, in Tables, the benefits of early treatment with senolytic drugs, for men and women.

In particular, women taking estrogen hormonal therapy and starting early treatment with senolytic drug can delay a state of osteoporosis by 10 years.

In this paper we focused on osteoporosis induced by senescence, and the effect of senolytic drugs in slowing the progression to osteoporosis. There are, of course, several standard drugs in the treatment of osteoporosis, such as Denosumab and BP Alendronate. It would be interesting to study therapy that combines such a drug with a senolytic drug (e.g., fisetin or quercetin) once the senolytic drugs have been successful in clinical trials.

Other comments

Following some comments that need to be revised.
1. Table 6 can be moved to the methods section and show earlier so that the readers know what each variable represents in the equation.

Response. We have moved Table 6 (now Table 2) in the Modeling section (Page 5).

2. Page 2, paragraph 4 “Senescence mesenchymal stem cell (MSC) derived exosomal microRNAs, include miRNA-34 [10–12] and miRNA-183-5p [10, 13, 14].” This sentence read like not a complete sentence.

Response. We have corrected this sentence, which now reads as follows: Senescence mesenchymal stem cell (MSC)-derived exosomal microRNAs include miRNA-34 [10–12] and miRNA-183-5p [10, 13, 14]. (Page 2, Paragraph 2).

3. Page 2: Check this sentence for accuracy "Immature MSCs are derived from bone marrow and their recruitment is enhanced by TGF-β [15]".

Response. We have rewritten this sentence and it now reads thus:
Immature MSCs are derived from bone marrow and their recruitment are regulated and can be enhanced by the TGF-β family [15,16]. (Page 2, Paragraph 4).

4. "The stability of the bone depends on the relative concentration of three proteins: RANK, RANKL and OPG." This statement appears not correct. Maybe "Bone remodeling depends. . . .". This needs a citation. These three factors regulate osteoclastogenesis or bone resorption.

Response. We thank the Reviewer for the correction. We have rewritten this sentence as follows:
Bone remodeling depends on the relative concentration of three proteins: RANK, RANKL and OPG, which regulate osteoclastogenesis or bone resorption [17]. (Page 2, Paragraph 5).

5. In term of the effect of fisetin on cell senescent and bone loss, reference 53 did not show fisetin had beneficial effects on bone loss of Z24 Progeria mice despite others showed it could decrease senescent cells in other model of progeria mice or normal aging mice (reference 57). So it maybe not be appropriate to use reference 53 as guidance for the mathematics modeling of fisetin.

Response. We thank the Reviewer for the comment and suggestion. We have now dropped the former reference [53] and have replaced it with references [71–73] (last paragraph in Page 12, in red).
