# Supplementary material for: Osteoporosis induced by cellular senescence: A mathematical model
Source: PLoS One. 2024 May 28;19(5):e0303978. doi: 10.1371/journal.pone.0303978 (PMC11132490; doi:10.1371/journal.pone.0303978)
Supplement: Attachment — Submitted filename: Comments to Plos one 1.docx [file pone.0303978.s001.docx]

Comments to Plos one

This is a very interesting paper. Authors attempted to consider positive and negative factors of bone formation and osteoporosis as well as cell senescence using mathematical modeling methods. The results were able to predict bone loss according to aging as well as benefit of the senolytic drug. However, I think the paper sections need to be reorganized according to Journal format. Some of the introduction parts can be moved to “methods and modeling” section. Figure 1 should be in modeling section and served as hypothesis of the mathematic modeling. The conclusion read like discussion. Authors should consider adding a discussion section and only put the summary of this paper as a conclusion.

Following some comments that need to be revised.

1. Table 6 can be moved to the methods section and show earlier so that the readers know what each variable represents in the equation.
2. Page 2, paragraph 4 “Senescence mesenchymal stem cell (MSC) derived exosomal microRNAs, include miRNA-34 [10–12] and miRNA-183-5p [10, 13, 14].” This sentence read like not a complete sentence.
3. Page 2: Check this sentence for accuracy “Immature MSCs are derived from bone marrow and their recruitment is enhanced by TGF-β [15]”.
4. “The stability of the bone depends on the relative concentration of three proteins: RANK, RANKL and OPG. This statement appears not correct. Maybe “Bone remodeling depends….”. This needs a citation. These three factors regulate osteoclastogenesis or bone resorption.
5. In term of the effect of fisetin on cell senescent and bone loss, reference 53 did not show fisetin had beneficial effects on bone loss of Z24 Progeria mice despite others showed it could decrease senescent cells in other model of progeria mice or normal aging mice (reference 57). So it maybe not be appropriate to use reference 53 as guidance for the mathematic modeling of fisetin.
